# Lipomatous tumors of adrenal gland: A case series of 5 patients and review of the literature

**DOI:** 10.1016/j.ijscr.2020.01.027

**Published:** 2020-01-26

**Authors:** Abdullah Mahmoud Alkhalifa, Mohammed Yousef Aldossary, Ammar Jamal Abusultan, Abdullah Saleh AlQattan, Mohammed Alsomali, Fatimah Alquraish, Njoud Alkhaldi, Jamal Y. Alsaeed, Abdulaziz M. Alwosaibei, Mohammed Alqambar, Osama Habib Alsaif

**Affiliations:** a Imam Abdulrahman Bin Faisal University, P.O. Box 1982, Al-Khobar, Dammam 31441, Saudi Arabia; b Department of General Surgery, Surgical Oncology Section, King Fahad Specialist Hospital-Dammam, Saudi Arabia; c Department of Pathology and Laboratory Medicine, King Fahad Specialist Hospital-Dammam, Saudi Arabia; d Department of General Surgery, King Abdulaziz Airbase Armed Forces Hospital, Dhahran, Saudi Arabia; e Department of Internal Medicine, Endocrine Section, King Fahad Specialist Hospital-Dammam, Saudi Arabia

**Keywords:** AML, angiomyolipomas, US, ultrasonography, CT, computed tomography, SCD, sickle cell disease, MRI, magnetic resonance imaging, EMH, extramedullary hematopoiesis, HMB-45, human melanoma black-45, Adrenal gland, Myelolipoma, Angiomyolipoma, Tumor

## Abstract

**Introduction:**

Lipomatous tumors of the adrenal glands are a diverse group of tumors characterized by the composition of adipose tissue. This group of tumors include myelolipomas, angiomyolipomas (AML), lipomas, and teratomas. They are usually benign, non-functioning tumors, and they account for 5% of all primary adrenal tumors. This study aimed to elucidate the management of the initial size of the adrenal mass and symptoms of presentation. We provide a succinct literature review regarding angiomyolipomas tumors of the adrenal glands.

**Presentation of cases:**

Here, we report 5 cases of lipomatous tumors of the adrenal glands. All 5 tumors were non-functioning. Four of them were myelolipoma, and one was AML. Two cases of myelolipoma presented with flank pain, while the rest of the 3 cases presented with hypochondrium pain. Three cases of myelolipoma were managed with laparoscopic adrenalectomy, one case of myelolipoma was converted from laparoscopic to open adrenalectomy because of abdominal adhesions, and the last case was AML and was managed with open adrenalectomy. All patients had an uneventful recovery with regular follow-up.

**Discussion:**

Myelolipoma, the most common lipomatous tumor of the adrenal gland, consists of a mixture of bone morrow element and adipose tissue. AML consists of a mixture of a thick-walled blood vessel, smooth muscle, and adipose tissue. They mimic many different benign and malignant tumors on radiography, and the histopathological examination is still needed to confirm the diagnosis. There is still controversy in the management of these tumors. Usually, the management is individualized on each case. Reporting of these tumors are increasing due the wide-spread use of modern imaging modalities.

**Conclusion:**

Adrenal lipomatous tumors are uncommon but with the increase use of imaging modalities their detection has increased. Further studies are needed to establish guidelines in the management of these tumors, especially that they can mimic malignant conditions.

## Highlights

• Lipomatous tumors of the adrenal glands are a diverse group of tumors that contain significant amount of adipose tissue.
• They are usually benign, non-functioning tumors but have some malignant types such as liposarcoma.
• Myelolipomas are the most common type of lipomatous tumors.
• Another rare entity of lipomatous tumors of the adrenals is angiomyolipomas.
• The management approach should be individualized for each case.

## 1 Introduction

Lipomatous tumors of the adrenal glands are a diverse group of tumors that contain significant amount of adipose tissue [1]. This group include myelolipomas, AML, lipomas, and teratomas [1]. They are usually benign, non-functioning tumors but have some malignant types such as liposarcoma [1]. They account for 5% of all primary adrenal tumors [1]. Myelolipomas are the most common type of lipomatous tumors of the adrenals, they consist of adipose tissue and hematopoietic tissue, and their incidence on autopsy is between 0.08 to 0.2% [2]. Another rare entity of lipomatous tumors of the adrenals is AML with an incidence of 0.13% [1]. Adrenal lipomatous tumors are usually asymptomatic and usually present as an incidental finding in the radiological images. However, they can present with symptoms such as flank pain, or with a more catastrophic presentation like retroperitoneal hemorrhage [3]. A definitive diagnosis cannot be reached by imaging studies alone, as these tumors can be very similar to each other and can be similar to other tumors as well. Therefore, the histopathological examination is needed to reach a definite diagnosis and to rule out malignancy [4]. Unfortunately, due to the rarity of these tumors, they have not been studied very well and still there is no guideline on how to manage these tumors. Therefore, the management should be individualized for each case [[5], [6], [7]]. We report five cases of adrenal lipomatous tumors, 4 of them were myelolipoma, and one was AML. This study aimed to elucidate the management of the initial size of the adrenal mass and the symptoms of presentation. We provide a succinct literature review regarding angiomyolipomas tumors of the adrenal glands. Imaging studies and histological characteristics are discussed, as well as surgical management. This case series has been reported according to surgical case series criteria [8].

## 2 Presentation of cases

### 2.1 Case 1

**Fig. 1:**
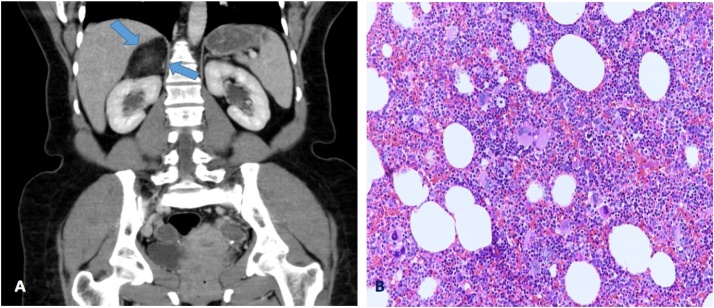
**A**: CT scan of the abdomen and pelvis revealed right adrenal mass measuring approximately 6 × 4 × 6 cm with predominant fat with patchy areas which suggestive of adrenal myelolipoma. **B**: Haemotoxylin and eosin staining (200×) a well-defined lesion composed predominantly of mature adipose tissue along with hematopoietic cells with the presence of unremarkable rim of adrenal tissue.

A 46-year-old female, with no known history of any medical illness, was referred to our surgical outpatient department with complains of mild to moderate intermittent abdominal pain in the right hypochondrium for 5 months. The patient denied any history of anorexia, weight loss, and jaundice. Ultrasonography (US) of abdomen was done in the referral hospital at that time and showed a large oval-shaped hypoechoic focal lesion superior to the right kidney. On physical examination, the patient was well-nourished, in moderate pain, and had no pallor or jaundice. Abdominal examination revealed mild tenderness in the right hypochondrium area with no palpable masses. Laboratory examination revealed the following: haemoglobin: 9.6 g/dl, leucocyte count: 6.06 × 10^9^/L, haematocrit: 30.3%, and platelet count: 359 × 10^9^/L. The liver function test, renal function test, and the coagulation profile were all within normal ranges. Hormonal profiles were all within normal ranges, including the investigations of serum cortisol, aldosterone, metanephrine, normetanephrine, 5 hydroxyindoleacetic acid, adrenocorticotropic hormone, and renin. Computed tomography (CT) scan of abdomen and pelvis revealed right adrenal mass measuring approximately 6 × 4 × 6 cm with predominant fat with patchy areas of heterogeneous attenuation with no evidence of enlarged lymph nodes or invasion to the surrounding organs and vessels. No other mases were found. The mass was circumscribed with well-defined borders which was suggestive of adrenal myelolipoma (Fig. 1A). Therefore, the patient underwent transperitoneal laparoscopic right adrenalectomy. Histopathological examination revealed a well-defined lesion composed predominantly of mature adipose tissue along with hematopoietic cells with the presence of unremarkable rim of adrenal tissue (Fig. 1B). The postoperative recovery was uneventful, and the patient was discharged home at post-operative day 2 with regular follow-up in our outpatient department clinic.

### 2.2 Case 2

**Fig. 2:**
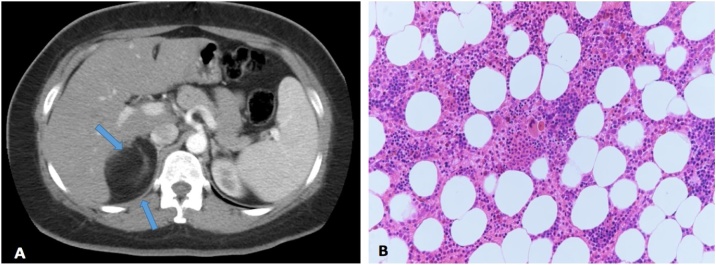
**A**: CT scan of the abdomen and pelvis revealed right adrenal mass measuring approximately 6 × 5 cm oval-shaped soft tissue mass lesion in the right adrenal gland. It showed fatty contents with thin internal septations. **B**: Haemotoxylin and eosin staining (200×) the tumor demonstrated predominantly mature and benign adipose tissue interspersed by trilineage hematopoietic components characteristics of myelolipoma.

A 35-year-old female, known to have diabetes mellitus, who’s found to have a tumor discovered incidentally on imaging during a workup for complicated urinary tract infection in the referral hospital 9 years ago. Since its discovery the patient didn’t seek any medical intervention, and she remained asymptomatic. However, 5 months ago the patient started to have an exacerbation of her right flank pain for which she underwent further workup, CT scan of abdomen and pelvis done at that time in the referral hospital and showed an interval increase in the size of the right adrenal mass (from 3 × 4.3 cm to 6 × 5 cm). The patient denied any history of anorexia and weight loss. Upon arrival at our hospital, the patient was well-nourished, in moderate pain, and had no pallor or jaundice. Abdominal examination revealed moderate tenderness in the right flank area with no palpable masses. Laboratory examination revealed the following: haemoglobin: 9.2 g/dl, leucocyte count: 6.24 × 10^9^/L, haematocrit: 31.2%, and platelet count: 249 × 10^9^/L. The liver function test, renal function test, and the coagulation profile were all within normal ranges. Hormonal profiles were all within normal ranges, including the investigations of serum cortisol, aldosterone, metanephrine, normetanephrine, 5 hydroxyindoleacetic acid, adrenocorticotropic hormone, and renin. CT scan of the abdomen and pelvis revealed right adrenal mass measuring approximately 6 × 5 cm oval-shaped soft tissue mass lesion in the right adrenal gland. It showed fatty contents with thin internal septations. No definite invasion of adjacent organs was detected. No other masses were found. The findings were suggestive for Adrenal Myelolipoma (Fig. 2A). Therefore, the patient underwent transperitoneal laparoscopic right adrenalectomy. Histopathological examination of the tumor demonstrated predominantly mature and benign adipose tissue interspersed by trilineage hematopoietic components characteristics of myelolipoma (Fig. 2B). The postoperative recovery was uneventful, and the patient was discharged home at post-operative day 3 with regular follow-up in our outpatient department clinic.

### 2.3 Case 3

**Fig. 3:**
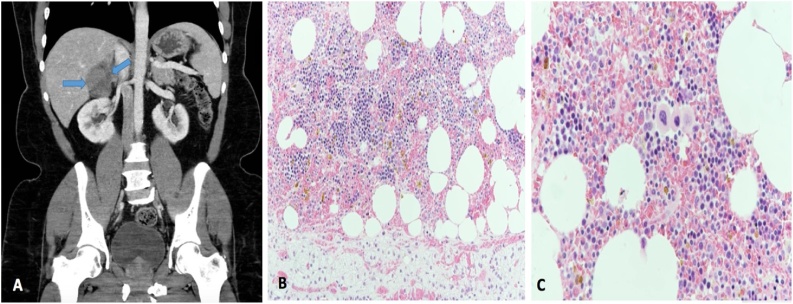
**A**: CT scan of the abdomen and pelvis revealed a horseshoe kidney with few small sub-centimeter cortical cysts on the right-side kidney, along with a 9 × 7 × 6.6 cm well-defined right adrenal mass of mixed density containing soft tissue enhancement and fatty component. **B**: Haemotoxylin and eosin staining (200×) trilineage hematopoietic components within mature adipose tissue attached to the histologically unremarkable adrenal cortex. **C**: Haemotoxylin and eosin staining (400×) with same findings.

A 45-year-old male, known to have hypertension. The patient was referred to our surgical outpatient department with complaints of mild to moderate intermittent pain in the right flank associated with dysuria and hematuria for 10 months. The patient denied any history of anorexia, weight loss, and jaundice. Past surgical history was remarkable only for previous exploratory laparotomy due to motor vehicle accident 6 years back. On physical examination, the patient was well-nourished, in mild pain, and had no pallor or jaundice. Abdominal examination revealed midline laparotomy scar with mild tenderness in the right flank area on palpation, and there were no palpable masses. Laboratory examination revealed the following: hemoglobin: 11.7 g/dl, leucocyte count: 4.18 × 10^9^/L, hematocrit: 35.7%, and platelet count: 189 × 10^9^/L. The liver function test, renal function test, and the coagulation profile were all within normal ranges. Hormonal profiles were all within normal ranges, including the investigations of serum cortisol, aldosterone, metanephrine, normetanephrine, 5 hydroxyindoleacetic acid, adrenocorticotropic hormone, and renin. CT scan of the abdomen and pelvis revealed a horseshoe kidney with few small sub-centimeter cortical cysts on the right-side kidney, along with a 9 × 7 × 6.6 cm well-defined right adrenal mass of mixed density containing soft tissue enhancement and fatty component. No other masses were found (Fig. 3A). The clinical and radiological pictures were suggestive for adrenal myelolipoma. The patient underwent transperitoneal laparoscopic right adrenalectomy converted to open exploratory laparotomy due to multiple adhesions of the previous laparotomy surgery. Histopathological examination revealed trilineage hematopoietic components within mature adipose tissue attached to the histologically unremarkable adrenal cortex (Fig. 3B and C). The postoperative recovery was uneventful, and the patient was discharged home at post-operative day 2 with regular follow-up in our outpatient department clinic.

### 2.4 Case 4

**Fig. 4:**
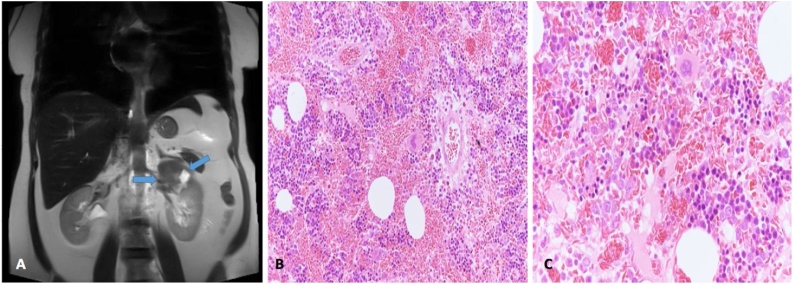
**A**: MRI of abdomen and pelvis revealed a well-defined mass measuring 7 × 6 cm arising from the lateral limb of left adrenal gland, associated with another well-defined nodule arising from the medial limb of the left adrenal gland as well measuring 1.5 × 2.5 cm. **B**: Haemotoxylin and eosin staining (200×) sections of the first mass revealed trilineage hematopoietic tissue with extravasated sickled red blood cells intermixed with benign adipose tissue. **C**: Haemotoxylin and eosin staining (400×) with same findings.

A 47-year-old female, known to have sickle cell disease (SCD), glucose-6-phosphate dehydrogenase deficiency, and cholelithiasis which was managed with cholecystectomy. The patient was found to have an incidental left adrenal mass during workup of moderate left flank pain for 4 years. The patient denied any history of anorexia and weight loss. Also, the patient denied any history of gastrointestinal and urinary symptoms. On physical examination, the patient was thin, in mild pain, and had no pallor. Abdominal examination revealed mild tenderness in the left flank area, and there were no palpable masses. Laboratory examination revealed the following: normocytic normochromic anemia, low calcium: 2.05 mmol/L, and high C-reactive protein: 11.6 mg/L, which is consistent with her sickle cell disease. Complete blood count revealed: hemoglobin: 9.6 g/dl, leucocyte count: 11.48 × 10^9^/L, hematocrit: 28.9%, and platelet count: 243 × 10^9^/L. Liver function tests demonstrated the following: albumin, 32 g/L, total protein: 63 g/L, alanine aminotransferase: 47 units/L, aspartate transaminase: 43 units/L, alkaline phosphatase: 88 units/L, total bilirubin: 16.27 μmol/L, and conjugated bilirubin: 7.4 μmol/L. Renal function test, and the coagulation profile were all within normal ranges. Hormonal profiles were all within normal ranges, including the investigations of serum cortisol, aldosterone, metanephrine, normetanephrine, 5 hydroxyindoleacetic acid, adrenocorticotropic hormone, and renin. Magnetic resonance imaging (MRI) of abdomen and pelvis was done in the referral hospital with no clear explanation of using this imaging modality. The MRI revealed a well-defined mass measuring 7 × 6 cm arising from the lateral limb of left adrenal gland. It demonstrated heterogeneous low to intermediate signal intensity on T1, low peripheral solid component on T2 with central high signal intensity representing necrosis/hemorrhage. There was another well-defined nodule arising from the medial limb of the left adrenal gland as well measuring 1.5 × 2.5 cm, it showed drop in signal intensity on in/out of phase in keeping with adenoma. There were no other masses detected (Fig. 4A). An extramedullary hematopoiesis (EMH) was considered in this case because the patient is a known case of SCD. Therefore, the patient underwent transperitoneal laparoscopic left adrenalectomy. Gross examination revealed: the first mass consisted of dark red hemorrhagic mass (6 × 5 × 4.5 cm) with yellowish areas. The other mass was well-circumscribed nodular (2.5 × 1.5 × 1 cm) at one limb of the adrenal gland with a golden yellowish cut surface reminiscent of adjacent adrenal cortex with friable center. On light microscopic examination, sections of the first mass revealed trilineage hematopoietic tissue with extravasated sickled red blood cells intermixed with benign adipose tissue (Fig. 4B and C). The differential diagnosis included EMH or myelolipoma, but the diagnosis of myelolipoma was rendered given the radiologic and gross findings of distinct mass.

The postoperative recovery was uneventful, and the patient was discharged home at post-operative day 2 with regular follow-up in our outpatient department clinic.

### 2.5 Case 5

**Fig. 5:**
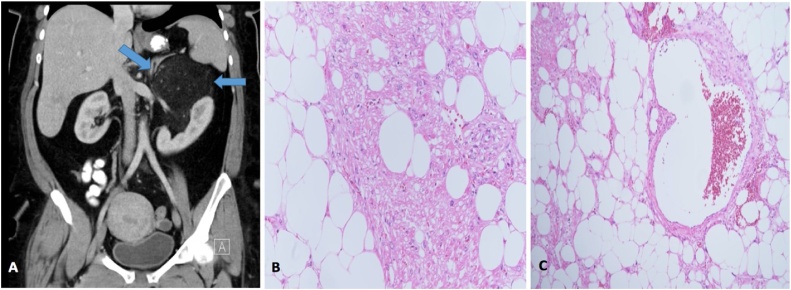
**A**: CT scan of the abdomen and pelvis revealed a large fatty mass measuring 16 × 11 × 10 cm on the left upper abdomen. It was displacing the left kidney inferiorly, laterally and partially encasing the medial aspect and the upper pole of left kidney. Small interspersed vascular branches from the left renal vessels noted along the margin and within the mass. **B**: Haemotoxylin and eosin staining (200×) the tumor composed of a mixture of mature adipose tissue, smooth muscle fibers, and aberrant dilated vasculatures. **C**: Haemotoxylin and eosin staining (100×) with same findings.

A 45-year-old female, known to have diabetes mellitus, hypertension, and iron deficiency anemia. The patient was referred to our surgical outpatient department with complaints of mild intermittent pain in the left hypochondrium area for 10 months. The patient denied any history of anorexia, weight loss, and jaundice. Past surgical history was remarkable only for sleeve gastrectomy 9 months ago. On general physical examination, the patient was well-nourished, in mild pain, and had no pallor or jaundice. Abdominal examination revealed mild tenderness in the left hypochondrium area with no palpable masses. Laboratory examination revealed the following: haemoglobin: 13.3 g/dl, leucocyte count: 7.35 × 10^9^/L, haematocrit: 43.7%, and platelet count: 221 × 10^9^/L. The liver function test, renal function test, and the coagulation profile were all within normal ranges. Hormonal profiles were all within normal ranges, including the investigations of serum cortisol, aldosterone, metanephrine, normetanephrine, 5 hydroxyindoleacetic acid, adrenocorticotropic hormone, and renin. CT scan of the abdomen and pelvis revealed a large fatty mass measuring 16 × 11 × 10 cm on the left upper abdomen. It was displacing the left kidney inferiorly, laterally and partially encasing the medial aspect and the upper pole of left kidney reaching to the level of the left renal vessels inferiorly and abutting the diaphragm superiorly. It was also displacing the limbs of the left adrenal gland and abutting the posterior surface of the spleen and the inferior surface of the pancreatic tail. Small interspersed vascular branches from the left renal vessels noted along the margin and within the mass (Fig. 5A). The radiological impression was AML versus liposarcoma. Therefore, the patient underwent CT guided biopsy for the adrenal mass which was negative for liposarcoma. Therefore, the patient was then prepared for the surgery with preoperative percutaneous trans-arterial angiographic embolization of the feeding vessels followed by exploratory laparotomy and en-bloc resection of the left adrenal gland. Gross examination revealed a mass containing predominantly lobulated homogenous yellow fatty cut surfaces displacing the adrenal gland. There were no areas of hemorrhage or necrosis. Microscopically, the tumor composed of a mixture of mature adipose tissue, smooth muscle fibers, and aberrant dilated vasculatures (Fig. 5B and C). Immunohistochemical stains showed that the neoplastic cells are positive for Human Melanoma Black-45 (HMB-45) and Melan-A. The histological and immunohistochemical findings supported the diagnosis of adrenal AML. The postoperative recovery was uneventful, and the patient was discharged home at post-operative day 5 with regular follow-up in our outpatient department clinic.

## 3 Discussion

Lipomatous tumors of the adrenal glands are usually benign, non-functioning tumors but have some malignant types such as liposarcoma [1]. It is important to recognize their features as their detection had increased in the last decade. It is probably due to the more spread use of imaging studies. However, their prevalence cannot be assessed correctly due to the fact that most of the patients are asymptomatic [9].

Myelolipoma of the adrenal gland usually consists of a mixture of bone morrow element and adipose tissue [9]. It is considered as the most common type of lipomatous tumors but It is rarely to be seen at other parts of the body [10].

The origin of Myelolipoma is believed to be due to a stimuli to the reticuloendothelial cells of the blood capillaries such as necrosis, infection or stress that lead to metaplastic changes [9]. Deccman et al. [9] reported in their review that there is no remarkable difference in male to female ratio, the average age of presentation is in the 50 s, and it is more commonly found in the right adrenal gland than the left [9]. In our case series, 3 out of 4 adrenal myelolipomas were on the right side which is consistent with their review. Myelolipoma can be similar to EMH. But they can be differentiated based on clinical, radiological, and histopathological features. Myelolipomas are associated with healthy bone marrow and they are well-demarcated lesions and contain adipose tissue. While EMH is associated with hematological conditions, and usually are not well-demarcated and adipose tissue is not usually seen [9]. In case 4, we considered both myelolipoma and EMH in the differential diagnosis as the patient is a known case of SCD, but the clinical features, radiological findings, and the gross examination findings are more consistent with myelolipoma. It has been reported that myelolipoma is associated with hypertension, atherosclerosis, diabetes mellitus, and other chronic diseases. As well as endocrine disorders such as congenital adrenal hyperplasia, Cushing’s syndrome, and Conn’s syndrome [9]. Interestingly, some patients had associated hormonal manifestations due to elevated steroids, aldosterone, sex hormones or catecholamine but after resection of the tumor, the manifestations disappeared [10]. Hence, the cause of hypersecretion was suggested to be due to mechanical irritation by the tumor [10].

On the other hand, AML is one of lipomatous tumor that is derived from perivascular epithelioid cells [1]. They consist of a variable proportion of thick-walled blood vessel, smooth muscle, and adipose tissue [11]. The remark of this tumor is the immunoreactivity of HMB-45 and muscle markers (desmin, actin, vimentin, keratin, epithelial membrane antigen) [7]. The average age of presentation is in the 40 s [7]. The most common sites for these lesions are the kidneys, followed by the liver [7]. AMLs of the adrenals are extremely rare with an incidence of 0.3–3% [12] with only a few reports in the literature [3].

Table 1 shows a summary of 28 cases of angiomyolipoma of the adrenal glands from 2001 to 2019 in the literature including one case from our case series. The first 2 cases of an angiomyolipoma of the adrenal glands were reported by Lam et al. in 2001 [10]. This summary shows a female predominate in which 19 cases of angiomyolipoma were females (67.9%). Only two cases were below 30 of age [10,28]. The age group ranges from 20 to 72 with a mean of 46.3. Eleven cases in the literature had angiomyolipoma in the left adrenal gland, 16 in the right, and 1 had bilateral angiomyolipoma. The most common initial presentation was localized abdominal pain in 15 cases, especially in the upper hypochondrium area (8 cases). followed by 7 cases as an incidental finding.

**Table 1:** Summary of 28 cases of angiomyolipoma of the adrenal glands from 2001 to 2019 in the literature including one case from our case series.

Patient	Reference	Age/gender	Presentation	Side	Size	Type of surgery
1	Lam et al. [14]	46 - F	Incidental finding	Left	CT: 8 cm in maximum diameter	Open left adrenalectomy
2	Lam et al. [14]	20 - M	Incidental finding at nephrectomy	Left	On specimen: 0.2 cm	Open left adrenalectomy with left radical nephrectomy
3	Elsayes et al. [15]	49 - F	Referred for MRI	Right	MRI: 12.2 × 9.8 × 6.8 cm	Surgical resection of the right adrenal mass (Type of the surgery was not specified)
4	Sutter et al. [16]	32 - F	Diffuse abdominal pain	Right	CT: 6 cm in maximum transverse diameter	Laparoscopic right adrenalectomy
5	Godara et al. [17]	45 - F	Epigastric discomfort	Left	US: 15 × 12 cm	Exploratory laparotomy with left adrenalectomy
6	D'Antonio et al. [18]	42 - M	Left flank and back pain	Left	CT: 6 × 4.5 cm	Exploratory laparotomy with left nephrectomy and adrenalectomy
7	Kong et al. [19]	61 - M	Left loin pain	Left	CT: 10 × 10 × 10.1 cm	Laparoscopic left adrenalectomy
8	Gupta et al. [20]	42 - M	Upper hypochondrium pain	Right	CT: 8 × 5.5 × 4.5 cm	Exploratory laparotomy with right adrenalectomy and cholecystectomy
9	Yener et al. [21]	45 - F	Right upper hypochondrium pain	Right	US: 5 × 6 cm	Laparoscopic right adrenalectomy
10	Honglin Hu et al. [22]	55 - F	Right upper hypochondrium pain	Right	US: 15 × 16 cm	Open right adrenalectomy
11	Monowarul et al. [23]	37 - M	Generalized jerking discomfort and weakness	Right	US: 5.4 × 4.5 cm	Exploratory laparotomy with right adrenalectomy
12	Goswami et al. [7]	43 - F	Right loin pain	Right	MRI: 9.5 × 8 × 2 cm	Laparoscopic right adrenalectomy
13	Zhao et al. [4]	47 - F	Incidental finding	Left	On specimen: 6 cm	Open left adrenalectomy
14	Zhao et al. [4]	70 - M	Incidental finding	Left	On specimen: 8 cm	Open left adrenalectomy
15	Li et al. [24]	53 - M	Left hypochondrium abdominal pain	Left	US: 9 × 6 cm	Laparoscopic left adrenalectomy
16	Hafeez et al. [25]	72 - F	Right hypochondrium quadrant pain	Right	CT: 9 × 8.9 cm	Open right adrenalectomy
17	Kwazneski et al. [26]	65 - F	Right hypochondrium abdominal pain	Right	US: 11.3 × 9.4 cm	Open right adrenalectomy
18	Obin Ghimire et al. [6]	36 - M	Incidental finding	Right	CT: 5.2 × 4 × 3.1 cm	Laparoscopic right adrenalectomy
19	Obin Ghimire et al. [6]	61 - F	Incidental finding	Right	CT: 8.6 × 9.5 × 8.1 cm	Open right adrenalectomy
20	Chunchu HH et al. [27]	45 - M	Incidental finding and associated accelerated hypertension	Right	CT: 6.67 × 6.29 cm US: 6.17 × 6.72 cm	Laparoscopic right adrenalectomy
21	Jaiman et al. [5]	45 - F	intermittent epigastric pain with repeated episode of non-bilious vomiting	Right	US: 16 × 14 cm	Exploratory laparotomy with right adrenalectomy
22	Anwar et al. [28]	27 - F	Left flank pain	Left	US: 9 × 5 cm	Targeted therapy with the mTOR inhibitor Everolimus (tradename: Afinitor)
23	Bastakoti et al. [3]	54 - F	Left flank pain	Left	CT: 8 × 7 × 6 cm	Laparoscopic left adrenalectomy
24	Duralska et al. [29]	35 - F	Nonspecific abdominal pain	Left	CT: 8.2 × 7.2 cm MRI: 7 × 6 × 9 cm	Open left adrenalectomy
25	Valeshabad et al. [11]	33 - F	Abdominal discomfort	Bilateral, more in the left side	CT: 7.3 × 6.9 cm (left side)	Left adrenalectomy with left partial nephrectomy
26	Antar et al. [1]	48 - F	Right flank pain	Right	CT: 7 cm in maximum diameter	Open right adrenalectomy
27	Goswami A [13]	43 - F	right hypochondrium abdominal pain	Right	CT: 16 × 10.1 × 9.5 cm	Exploratory laparotomy with right adrenalectomy
28	Presented case.	45 - F	Right hypochondrium pain	Right	CT: 16 × 1 × 10 cm	Exploratory laparotomy with En-block resection of the left adrenal mass

Adrenal lipomatous tumors are usually asymptomatic and present as incidentaloma. However, they can present with flank/back pain, or with more catastrophic presentations such as retroperitoneal hemorrhage as a result of spontaneous rupture [3]. These symptoms usually occur as a result of compression of adjacent organs due to enlargement of the mass or when intra-tumor hemorrhagic necrosis develops [4].

In this series, 3 of our patients had hypochondrium pain in addition to dysuria in one patient, and the other two cases were incidental findings.

Imaging studies play an important role in the diagnosis of lipomatous tumors of the adrenal gland, but in some cases, it is challenging to distinguish a lipomatous tumor from the other types by imaging alone due to their similarities in these studies, as in AML tumors that contain small amount of fat. Also, malignancy cannot be ruled out completely. So, the histopathological examination is required to make the definitive diagnosis [4,30].

US is an effective initial tool for the detection of the mass size and its characteristics, but CT and MRI are more sensitive and aid in preoperative planning. High-fat content is pathognomonic. US can show a hyperechogenic mass on greyscale due to the fat component. CT scan usually demonstrates negative Hounsfield value, due to the fat component. However, the fat component is best visualized on MRI by using fat-suppression techniques [4]. Also, adrenal AML may present with central calcification and compression of the inferior vena cava [6]. Also, the thick-walled vessel component of AML can be seen on imaging as aneurysmal dilation [27].

In the gross examination of the myelolipoma and AML, they appear as well-demarcated tumors. They range from yellow appearance, to red and yellow, to more white-tan, respectively. While in the histological examination, myelolipoma shows a mixture of adipose tissue and hematopoietic elements such as myeloid, erythroid, and megakaryocytic lines. On the other hand, AML typically shows a variable mixture of a thick-walled blood vessel, spindled and epithelioid stromal cells and adipose tissue [5,10]. AML can be challenging to the pathologists, especially in cases where one tissue predominates. They are often misdiagnosed as lipoma, myelolipoma, liposarcoma, sarcoma, sarcomatoid carcinoma, or carcinoma. A diligent search for all the three tissues may help in confirming the diagnosis [3,17]. AMLs typically express melanocytic markers by immunohistochemistry including HMB-45, Melan-A, among others. They are variably positive for smooth muscle markers such as desmin and smooth muscle actin immunohistochemical stains 10]. It was difficult to determine the type of tumor in our patient in case 5 with imaging alone, and one of the top differential diagnosis was liposarcoma, but after the biopsy, we confirmed the diagnosis of AML. However, retroperitoneal liposarcoma represents 35–40% of all retroperitoneal sarcomas, with a classical presentation of non-specific symptoms secondary to the compression effect of the tumor on the surrounding tissues in most of the cases. The only therapeutic hope of liposarcomas is surgical resection, which is usually performed by open techniques, when possible, depending on the histological type which ranges from well-differentiated liposarcoma to dedifferentiated liposarcoma. The use of the laparoscopic technique in the treatment of liposarcoma is not widely used with only a few reported cases in the literature, all of which were for the treatment of well-differentiated type. However, Agrusa et al. reported the first case in the literature in which compartmental laparoscopic approach was performed for the treatment of a dedifferentiated case of liposarcoma to reduce the chances of local recurrences and increase disease-free survival years [31].

Although most of these tumors are non-functional, assessment of the functional status is very important [4]. All of our patients, fortunately, had non-functioning tumors.

There is still no guideline on how to manage these tumors. So, the management should be based on an individualized manner for each case. Based on previous experiences in the literature, management should be conservative for small, asymptomatic mass with follow up over 1–2 years with imaging studies. Surgical excision or arterial embolization is indicated if the patient is symptomatic, the tumor size is large or with enlarging tumor due to the risk of hemorrhage and malignancy [[5], [6], [7]]. There were different recommendations about the size of the tumor that should be considered for surgery, they range from 3.5–6 cm [4]. However, laparoscopic removal of the tumor can be done for larger tumors as well as in Castillo et al. did a laparoscopic adrenalectomy to mass measuring 14 cm without any complications [32].

Laparoscopic adrenalectomy has been recommended due to lower mortality compared with open surgery even with tumors larger than 5 cm [[5], [6], [7]].

Myelolipoma and AML are usually benign diseases with an excellent prognosis. However, a long follow-up period is necessary due to the uncertainty of the clinical progression of these rare tumors. Currently, there is no guideline for follow-up. Based on the published reports, an abdominal ultrasound at a time interval of 3–6 months following the surgery with an annual clinical examination of large tumors is recommended [4].

## 4 Conclusion

Adrenal lipomatous tumors are uncommon but with the increasing use of imaging modalities their detection increased. They are usually asymptomatic benign non-functioning tumors but they have malignant types and can be confused with other malignant masses. Hence, it is important to diagnose them correctly and follow them up to prevent any future complications. Also, a guidelines are needed for the management of these tumors.

## Funding

This study did not receive any funding from governmental or private organizations.

## Ethical approval

Ethical approval was obtained from the Institutional Review Board of the King Fahad Specialist Hospital, Dammam, Saudi Arabia. Reference number (SUR0334) Dated 22/10/2019.

## Consent

Written informed consent was obtained from the patients for publication of this case series and accompanying images. A copy of the written consent is available for review by the Editor-in-Chief of this journal on request.

## Author contribution

Study concept or design – MYD, ASQ, OHS.

Participation in the pre/post-operative management of the patients – NA, JYA, AMA, MA.

Data collection – MYD, AMK, MS, FQ.

Data interpretation – MYD, AMK, ASQ, AJA, FQ.

Literature review – AMK, ASQ, AJA.

Drafting of the paper – MYD, AMK, AJA, ASQ.

Editing of the paper – MYD, ASQ, OHS.

## Registration of research studies

Researchregistry5168.

## Guarantor

Mohammed Yousef Aldossary.

## Provenance and peer review

Not commissioned, externally peer-reviewed.

## Declaration of Competing Interest

The authors declare no conflict of interest
